# Slice & Dice: nested spin–lattice relaxation measurements†

**DOI:** 10.1039/d2cp03458a

**Published:** 2022-10-12

**Authors:** W. Trent Franks, Jacqueline Tognetti, Józef R. Lewandowski

**Affiliations:** a Department of Chemistry, University of Warwick Coventry CV4 7AL UK J.R.Lewandowski@warwick.ac.uk; b Department of Physics, University of Warwick Coventry CV4 7AL UK

## Abstract

Spin–lattice relaxation rate (*R*_1_) measurements are commonly used to characterize protein dynamics. However, the time needed to collect the data can be quite long due to long relaxation times of the low-gamma nuclei, especially in the solid state. We present a method to collect backbone heavy atom relaxation data by nesting the collection of datasets in the solid state. This method results in a factor of 2 to 2.5 times faster data acquisition for backbone *R*_1_ relaxation data for the ^13^C and ^15^N sites of proteins.

## Abstract 2

Slice & Dice experiment accelerates up to 2.5 times measurements of spin–lattice relaxation rates for characterizing protein dynamics by nesting ^13^C and ^15^N experiments.

## Graphical abstract

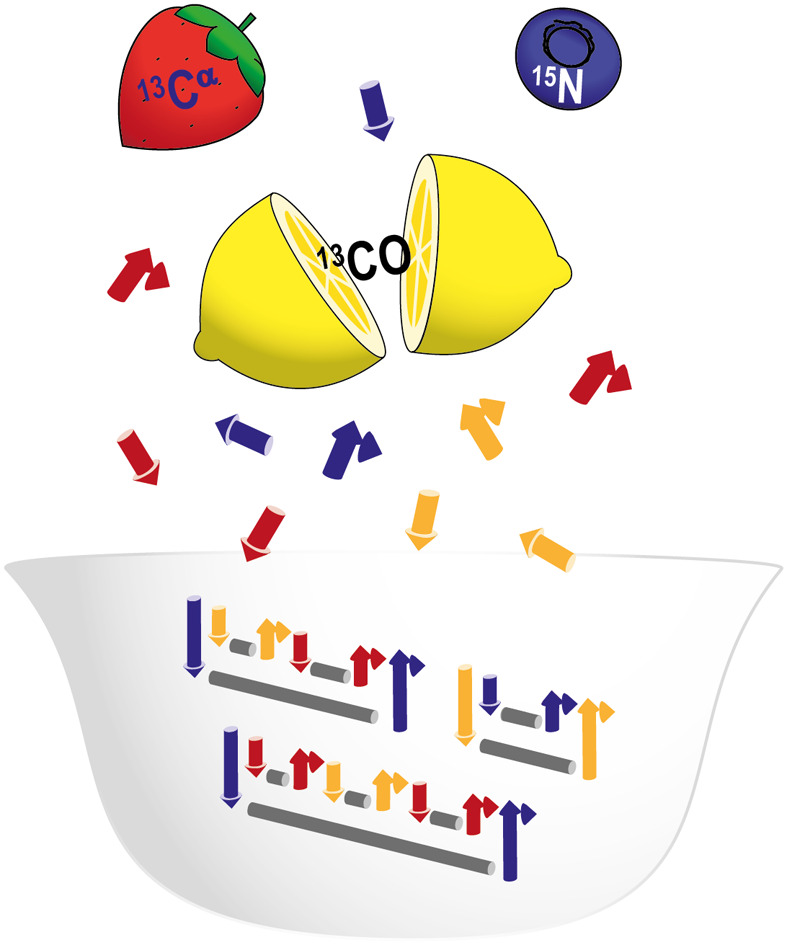

## 1 Introduction

One of the strengths of Nuclear Magnetic Resonance (NMR) is that it can probe molecular motions under near physiological conditions in which the only perturbation is labelling with NMR-active isotopes. NMR relaxation measurements are commonly employed to probe time scales and amplitudes of molecular motions at atomic resolution.^1,2^ In the solid state, in the absence of the overall tumbling, the time scale window that can be observed is expanded compared to solution, and measurements could be performed in even very large systems. For example, local dynamics could be studied in large protein complexes that are amenable to structural characterization only *via* cryo-EM. However, since each individual relaxation rate samples only a limited range of frequencies, multiple measurements are typically required to reasonably constrain the motions. To increase the range of sampled frequencies and improve the description of dynamics, measurements are performed at different magnetic fields, different temperatures and for different nuclei.^3–5^ This contributes to relaxation measurements being generally time-consuming experiments.

Longitudinal relaxation rates (*R*_1_ = 1/*T*_1_)^6^ report on motions with correlation times in the order of ps–ns. In the solid state, backbone ^13^C and ^15^N nuclei are typically characterized by long *T*_1_ times in the order of tens of seconds. It is common to measure the relaxation in biological systems using pseudo-3D experiments^6–10^ in which site resolution is achieved from a 2D correlation spectrum, and the relaxation is encoded in the third, pseudo-dimension. The delays in this third pseudo-dimension are dictated by the length of *T*_1_s, which renders the experiments for probing backbone relaxation very long.

There have been several approaches to speed up the direct collection of *R*_1_ relaxation data, usually by partitioning the signal so that only one scan is needed. Single scan methods to measure *T*_1_ were first demonstrated by Kaptein *et al.*^11^ and later adapted using magnetic resonance imaging techniques (MRI).^12–14^ These techniques need very sensitive samples with detection on ^1^H or on hyperpolarized nuclei^15^ such as ^13^C or ^15^N. The high sensitivity is required due to signal splitting, along with good chemical shift resolution for site resolution and powerful gradients, which are all uncommon in biological NMR, and especially so for MAS experiments. Other approaches focus on speeding up acquisition by recording several experiments at once (PANACEA,^16^ DUMAS^17^), utilizing orphaned polarization,^18^ encoding multiple pathways into the same experiment,^19^ using multiple detectors,^20–22^ and by interleaving experiments into the recovery delay of another.^23^

We recently introduced experiments to collect protein backbone ^13^C′ and amide ^15^N relaxation data with a single excitation and sequential acquisitions.^24^ Our previous work presented simultaneous cross polarization (SIM-CP)^25^ and staggered acquisitions to encode carbon and nitrogen relaxation experiments using a shared time period. The ^13^C′ and ^15^N *R*_1_ rates are collected in the time it would normally take for the ^15^N *R*_1_ experiment. Here we present a method to acquire *R*_1_ relaxation measurements on three nuclei of the peptide plane ^13^C^α^, ^13^C′ and ^15^N through nested experiments, with no sensitivity loss, in the time normally required to acquire just a ^15^N *R*_1_ experiment.

## 2 Experimental methods

The T2Q mutant of GB1 was prepared with uniformly [^1^H,^13^C,^15^N] isotope enrichment as described previously^26^ and doped with 4,4-dimethyl-4-silapentane-1-sulfonic acid (DSS) as an internal standard. Approximately 0.5 mg of hydrated microcrystalline protein was packed into a 0.7 mm solid-state NMR rotor by centrifugation. The experiments were carried out on a Bruker a 0.7 mm HCND ultrafast MAS probe in triple resonance (HCN) mode at 700.13 ^1^H Larmor Frequency with a Bruker Avance III spectrometer. The sample was spinning at 100 kHz ± 3 Hz and was at a nominal temperature of 281.2 K (based on external calibration, calculated by the difference between the water and DSS peaks^27^) under a gas flow of 400 L h^−1^. The ^1^H RF carrier was placed at the center of the water resonance at 4.5 ppm, while the ^15^N was centered at 120 ppm. The ^13^C carrier was placed at 55 ppm for the alpha (^13^C^α^) and aliphatic (^13^C^ali^) carbons and at 175 ppm for the carbonyl carbons (^13^C′). The carbon carrier frequency was moved within the experiment using pre-determined constants to change the frequency. Each ^1^H FID was acquired for 30 ms, with a spectral width of 35 ppm with 16 coadded transients. ^13^C^ali^, ^13^C′ and ^15^N dimensions of the Slice & Dice and the standard ^13^C^ali^ experiment, were acquired with 64 increments each. The ^13^C^ali^ dimension was acquired with a dwell of 175 μs, with a spectral width of 32 ppm, for a total of 5.6 ms in the indirect dimension. Both ^13^C′ and ^15^N dimensions were acquired with a dwell of 300 μs for a total of 9.6 ms in the indirect dimension, and a spectral width of 19 ppm for ^13^C′ and 47 ppm for ^15^N. For the ^13^C′ standard measurement 72 increments were acquired with a dwell of 300 μs for a total of 10.8 ms in the indirect dimension. The ^15^N standard measurement was acquired with 84 increments in the indirect dimension with a dwell of 300 μs for a total of 12.6 ms in the indirect dimension. The States-TPPI method was employed for quadrature detection in the indirect dimension.^28^ The recovery delay was 1.5 s for all the experiments and the wait time (see Table S1, ESI†) was 1.5 s.

The nutation frequencies were calibrated for ^1^H at 2 μs (*ν*_1_ = 125 kHz), ^13^C at 2.5 μs (*ν*_1_ = 100 kHz) and ^15^N at 4.15 μs (*ν*_1_ = 60 kHz). Heteronuclear ^1^H decoupling (≈10 kHz WALTZ-64)^29^ was applied during ^13^C and ^15^N *t*_1_ evolution and during the COSY-based transfer. ^13^C heteronuclear decoupling (≈10 kHz WALTZ-64) was applied during the acquisition of both ^13^C experiments, while ^15^N heteronuclear decoupling (≈10 kHz WALTZ-64) was used only for the HN acquisition. The MISSISSIPPI^30^ solvent suppression scheme was applied with a spinlock field of ≈50 kHz for four 10 ms intervals after the excitation and chemical shift encoding period (*i.e.* immediately after storing the polarization along the *z*-axis) and for four 20 ms intervals immediately before transfer back to the ^1^H for detection for each individual *R*_1_ experiment. Cross-polarization (CP) was used for the initial excitation of ^13^C and ^15^N and the transfer back to ^1^H for acquisition. For all the experiments the average ^1^H field was chosen at ≈130 kHz with a linear 15% ramp (85–100%, from ≈121.5 to 139.5 kHz) and a zero-quantum (ZQ) match condition transfer was used on ^13^C and ^15^N channel. Each ^13^C and ^15^N frequency was irradiated at a field of ≈30 kHz and the carrier placed on the appropriate resonance. The contact times were optimized individually for the ^1^H–X/Y CP. The CP contact times were 1.2 ms, 2.1 ms and 2 ms for ^13^C^ali^, ^13^C′ and ^15^N respectively, while they were 150 μs for the one-bond ^13^C^ali^–^1^H transfer and 500 μs for ^15^N–^1^H CP. Gaussian Q3 cascade pulses were used for the selective ^13^C inversion where a 320 μs pulse gives a bandwidth of 10.5 kHz (≈60 ppm) and 760 μs gives a bandwidth of 5.3 kHz (≈30 ppm) for ^13^C′ and ^13^C^α^ respectively. For the selective ^13^C′–^13^C^α^ coherence transfer, the *J*-coupling delay was 4.25 ms when ^13^C′ is along the transverse plane and 3 ms when ^13^C^α^ is transverse.

The program used for arranging the experiments in the Slice & Dice experiments of the *R*_1_ was created in Python 3.7. The minimum and maximum relaxation times and the desired number of points for each sub-experiment can be entered manually or spaced automatically where the Fibonacci sequence is the basis for the spacing between time points. The pulse sequences, datasets, lists, compound pulse lists, and pulse shapes can be found online on Zenodo (https://doi.org/10.5281/zenodo.5965022). The relaxation delays used in the presented experiments are given in Table S1 (ESI†). To allow for direct comparison of the relaxation rates, the same number of increments in the indirect dimension were considered for all spectra. All spectra were processed in Bruker Topspin 3.6.1 with −40 Hz LB in the direct dimension and Lorentzian to Gaussian line broadening with −20 Hz and an offset of 0.1 in the indirect dimensions. Peak assignment and integration were performed using CARA version 1.9.1.7. The integrated intensities of each well-resolved peak were normalized and fit to a single exponential to find the relaxation rate. All relaxation rates are reported at the 95% confidence level from 2000 steps of Monte Carlo error analysis.^31^

## 3 Results and discussion

The vast majority of instrument time is spent waiting for the longest time points of the relaxation curve. There can be up to a ≈15 s delay between the acquisition for the ^13^C′ pathway and the acquisition for the ^15^N pathway. Taken to the logical extreme, one nucleus could be prepared and allowed to relax, but during its relaxation time a series of experiments could be run on a separate pathway that does not involve the original nucleus. Our previous work demonstrates that the rates measured using staggered acquisition reproduce the rates from standard experiments.^24^ Since this is the case, we concluded that the water suppression, ^13^C–^13^C COSY transfer, and ^13^C–^1^H inverse CP do not detectably perturb the spin dynamics on the stored ^15^N polarization. In the present work we extend this to the case of multiple embedded excitations and acquisitions leading to accelerated simultaneous measurement of longitudinal relaxation for several types of nuclei.

**Fig. 1:**
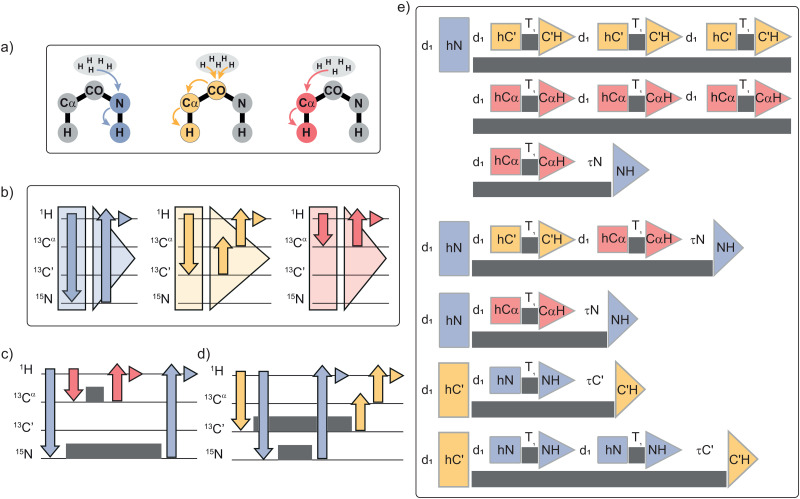
Schematic representation of the Slice & Dice implementation with (a) step-by-step magnetization transfer on the protein backbone. The nucleus involved in the *R*_1_ measurement is highlighted with a different colour for ^15^N (blue),^13^C′ (yellow), ^13^C^α^ (red), this colour coding is employed throughout the figure. (b) Slicing of the individual experiments in separate periods for excitation (square) and acquisition (triangle) which includes the back-transfer to proton and acquisition. Arrows display magnetization transfer and small triangles portray the acquisition period. (c) Representation of magnetization pathway when ^15^N acts as the “outer” experiment and (d) when ^15^N is the “inner” experiment. Grey squares display *T*_1_ periods and associated pulses. (e) Example of the Slice & Dice experiment ordering where coloured squares illustrate the preparation times as indicated, and triangles represent the back-transfer to proton and acquisition following the scheme in (b). In the panel (e) d_1_, *τ*N and *τ*C′ indicate respectively: relaxation delay, remaining time for ^15^N relaxation delay and remaining time for ^13^C′ relaxation delay. *τ*N and *τ*C′ ensure the correct relaxation delay times for “outer” experiments when the “inner” blocks do not fit perfectly.

Measurement of relaxation rates for different types of nuclei aids obtaining a more accurate view of protein motions. In this context one could measure the amide ^15^N, carbonyl ^13^C′, alpha carbon ^13^C^α^ and, if possible, the sidechain aliphatic carbons ^13^C^ali^s. The experiment time could be optimized by including the spin–lattice relaxation measurements on ^13^C^α^ and ^13^C′ with ^15^N *R*_1_s in an experiment we refer to as Slice & Dice. The magnetization transfer pathway for each nucleus ^15^N, ^13^C′ and ^13^C^α^ is illustrated in Fig. 1(a). The individual experiments are sliced into separate periods for excitation (square) and acquisition (triangle), as in Fig. 1(b). The excitation portion of the experiment has an initial CP, the chemical shift evolution and a storage pulse (Fig. S1, ESI†). With the use of a standard CP, where polarization is transferred to one type of nuclei, instead of a SIM-CP (where polarization is transferred to two types of nuclei), the excitation portion is treated separately for all the nuclei during the Slice & Dice construction. The acquisition portion of the experiment re-excites the stored polarization and then transfers it to the expected detection nucleus. The division of the pulse sequences into different blocks (indicated with squares and triangles) allows us to assemble them in the most convenient way to fit into the relaxation measurements as described below. In order to embed the blocks, we will only consider placing whole “inner” experiments into the relaxation delay of an “outer” experiment (Fig. 1). Generally, the ^15^N experiment requires the longest maximum relaxation delay, and the aliphatic carbons the shortest relaxation delay, so the ^15^N block will be made to be the first “outer” experiment, and the two ^13^C experiments will be the first “inner” experiments (Fig. 1(c)). The nitrogen relaxation measurement is the “outer” experiment for as long as the carbon “inner” experiments will fit into its relaxation delay. The “inner” and “outer” experiments are then exchanged when the long relaxation times of ^13^C are suitable to accommodate the short relaxation times of ^15^N that are now the “inner” experiment (as in Fig. 1(d)). In order to efficiently fit the experiments into one another, the order of the relaxation delays can be changed. It is usually possible to find a solution in which all or most of the desired relaxation times embed into one another nicely by hand, but can be a time-consuming puzzle, so a Python program was written to facilitate the creation of the experiments (see Data availability). The program will embed the experiments taking into account for the “AQ” time which includes recovery delay, excitation and evolution, 2× saturation and acquisition, and the “wait” time which ensures a minimum time between acquisitions.

The result of the ordering is an experiment similar to the one found in Fig. 1(e). There are 7 separate ^13^C experiments during the first ^15^N relaxation measurement, and then 2 and 1 in the relaxation delay of the next two ^15^N experiments. Then there are two ^13^C′ “outer” experiments with 1 and 2 ^15^N experiments embedded respectively. The provided Python program is used to estimate the timings for these experiments. To compare the experiment times, the time is estimated on the relaxation delay list without accounting for second chemical shift dimensions or for the repetitions needed for the phase cycle, then assuming that each experiment requires the same number of scans in total. For example, for crystalline GB1, considering an “AQ” time of 2.6 s, a “wait” time of 1.5 s, and the three delay lists in Table S1 (ESI†) the Python pulse program calculated that acquiring one transient for all these datasets in the traditional way would take 157.1 s for the ^15^N dataset, 62.7 s for the ^13^C^α^, and 101.2 s for the ^13^C′, or 321.0 s total, while it only takes 181.3 s for the embedded experiment. With the same delay list per nucleus between the usual implementation and Slice & Dice, and involving 16 transients and 64 indirect increments, the standard measurements would be 3 days and 8 hours (∼41 hours for ^15^N, ∼14 hours for ^13^C^α^ and ∼25 hours for ^13^C′), while the experimental time for Slice & Dice was ∼2 days and 2 hours.

Three sets of spin–lattice relaxation measurements for ^15^N, ^13^C′ and ^13^C^α^ are then acquired in approximately half the time necessary to collect the full complement of standard *R*_1_s, and if compared to the sole ^15^N standard experiment it takes only 8 h more. Alternatively, a standard ^13^C^α^ and a SIM-CP N + C′ experiment can be acquired separately. In this case, the Slice & Dice implementation takes 20% less time to collect. Further, the SIM-CP experiments suffer from ≈10% lower sensitivity while the interleaved experiments experience no loss since the Slice & Dice employs a standard CP for all of the ^1^H–X/Y transfer (Fig. S2, ESI†). It was found to be necessary to add a short “MISSISSIPPI” saturation period at the end of the excitation periods to ensure that the initial ^1^H polarization is consistent amongst all possible combinations of experiments and relaxation times.

**Fig. 2:**
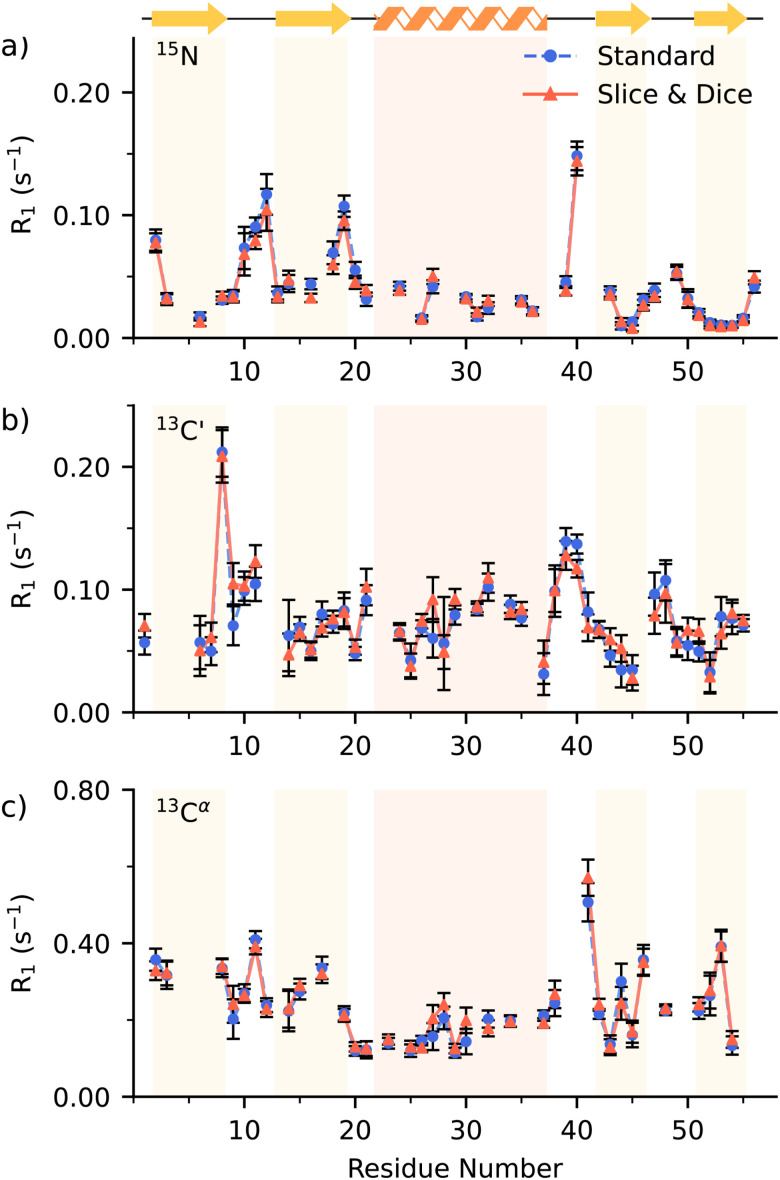
A comparison of the *R*_1_ rates for (a) ^15^N, (b) ^13^C′, (c) ^13^C^α^ obtained from the separated single-acquisition experiment (full-blue circle) and Slice & Dice (full-red triangle) as a function of the residue number. Errors bars represent two standard deviations within the correspondent rate. The shaded areas in panels (a)–(c) outline secondary structure elements in GB1 as indicated with cartoons above the panel (a). For the severely overlapping peaks, values are not included (see ESI†).

To test our experiments we used a fully protonated uniformly [^13^C,^15^N] enriched crystalline GB1 sample. In solid-state NMR the presence of spin diffusion alters the *R*_1_ rates, losing their site-specific nature due to averaging of nearby sites. In uniformly [^1^H,^13^C,^15^N] labelled samples at MAS > 20 kHz it is possible to obtain site specific rates on ^15^N,^32,33^ while spinning rates > 60 kHz are required for ^13^C′ *R*_1_s.^3,6^ All our experiments were carried out on a 700 MHz ^1^H Larmor frequency and a spinning frequency of 100 kHz. 100 kHz MAS guarantees truncation of proton driven spin diffusion on ^13^C^α^ of protonated uniformly labelled sample,^34^ which allows for the interpretation of *R*_1_ measurements at these sites. At lower spinning frequencies custom labelling schemes are required to minimize the relaxation rates averaging effects of the spin diffusion.^35^ Further, the fast MAS preserves the site-specificity bearing an improved ^1^H detected spectra resolution. The *R*_1_ measurements for ^15^N, ^13^C′ and ^13^C^α^ obtained with a single interleaved experiment are here compared with the rates recorded with the following separate acquisitions: ^15^N (Fig. 2(a)), ^13^C′ (Fig. 2(b)), and ^13^C^α^ (Fig. 2(c)), where the whole assignment can be found in ESI† (Fig. S3 (^13^C′, ^15^N) and S4 (^13^C^α^)). The rates are the same within measurement error to the standard implementation (see Fig. S7–S9 for correlation plots for the rates). The ^13^C^α^ measurement was set up to accommodate the acquisition of the aliphatic ^13^C, indeed the carbon dimension is folded at ≈43 ppm to divide the ^13^C^α^ resonances from the rest of the aliphatic carbons (Fig. S4 and S5, ESI†). The complete ^13^C^ali^ indirect dimension is incidentally acquired during the collection of the ^13^C^α^ measurements obtaining a well resolved sidechain ^1^H detected spectrum which may be feasible for relaxation measurements. However, 100 kHz MAS is still often not sufficient to fully average out spin diffusion on ^13^C^ali^ on an uniformly [^1^H,^13^C,^15^N] labelled sample^34^ and alternate ^13^C-labelling should be applied to minimise the effect of spin diffusion. For completeness, the comparisons between standard and Slice & Dice aliphatic ^13^C, ^13^C^β^ to ^13^C^α^, spin–lattice relaxation rates are reported in Fig. S6 (ESI†).

One of the challenges for these experiments is that the sampling of the indirect dimensions is linked to one another. The spectral width needed for the aliphatic ^13^C, or even the ^13^C^α^, is two to four times that needed for the ^13^C′ or ^15^N, depending on how the spectrum is folded. This discrepancy creates some relatively difficult decisions with respect to the sampling of the aliphatic fingerprint spectrum. In this sample there is a convenient place for folding the spectrum, but still the indirect ^13^C^α^ dimension is only sampled to about half the digital resolution of the other two spectra. This causes the resolution to be worse in the more crowded spectrum, which is not an ideal situation. It should be possible to extend the chemical shift evolution or even implement non-uniform sampling by calculating the timings and phases necessary. The sampling of the ^13^C^α^ chemical shift dimension could also be extended so that the experiment is collected in any number of blocks containing different increments for the indirect dimension, and then each portion concatenated onto the last, where the relaxation time is repeated the appropriate number of times for packing of the experiments. The method used here to split and rearrange the experiments should be valid under different experimental conditions. For example, in triply labelled [^2^H, ^13^C, ^15^N] and back-exchanged samples at slower spinning, the amide proton may be used exclusively as the read-out nucleus since the CP is efficient and fairly predictable amongst the three backbone nuclei: the amide, alpha carbon and carbonyl carbon. In this case, the COSY transfer in the ^13^C′ experiment would be removed in favor of a simple CP back to the amide proton. For site specific *R*_1_ measurements the aliphatic carbons could be made accessible at 50/60 kHz MAS through a combination of alternate ^13^C labelling and extensive deuteration,^35^ and at > 80 kHz with alternate ^13^C labelling.^34^

## 4 Conclusions

In summary, we demonstrate a strategy to utilize the instrument time more thoroughly for the collection of longitudinal relaxation experiments. We presented an approach to interleave the collection of *R*_1_ datasets for three sets of data ^15^N, ^13^C′ and ^13^C^α^ with no loss in sensitivity, and a decrease in the data collection time of 2 to 2.5 times that of the standard experiments. Further development of interleaved relaxation measurements could involve the creation of a higher dimensional experiments to improve the resolution in solid-state. Potentially it could be possible to obtain a 3D spectrum for the ^13^C. Heavily overlapping peaks on these resonances could then be potentially deconvoluted obtaining an even more complete picture of dynamics, especially considering the application of the Slice & Dice on large proteins and complexes.

## Data availibility

The raw NMR data for the experiments discussed in this manuscripts, pulse program and Python script for generating custom nested experiments, Python script to generate a macro to process and sort 2D planes of the Slice & Dice experiment, explanations on how to process Slice & Dice experiments are freely available on Zenodo: https://doi.org/10.5281/zenodo.5965022.

## Author contributions

WTF: developed original concept and methodology. Conducted experiments. Wrote software and original draft of the manuscript. JT: supported development of the concept. Conducted experiments. Processed and analysed the data. Supported writing software. Wrote original draft of the manuscript. JRL: supported development of the concept. Supervised the research. Edited manuscript.

## Conflicts of interest

The authors declare no conflicts of interest.

## Supplementary Material

CP-025-D2CP03458A-s001
