# Supplementary material for: Slice & Dice: nested spin–lattice relaxation measurements
Source: Phys Chem Chem Phys. 2022 Oct 12;25(8):6044–9. doi: 10.1039/d2cp03458a (PMC9945929; doi:10.1039/d2cp03458a)
Supplement: CP-025-D2CP03458A-s001 [file CP-025-D2CP03458A-s001.pdf]

Electronic Supplementary Material (ESI) for Physical Chemistry Chemical Physics.
This journal is © the Owner Societies 2022

Table S1. Delay lists for ^{15}N, $^{13}C'$ and $^{13}C^{\alpha}$ and R_1 measurements used in the standard and Slice & Dice experiments. Highlighted in grey the times used for the analysis which correspond to the initial part of the relaxation slope until the ~60% decay of the signal.

^{15}N (s)		$^{13}C'$ (s)		$^{13}C^{ali}$ (s)
Standard	Slice & Dice	Standard	Slice & Dice	Standard/Slice & Dice
0.25	0.01	0.10	0.01	0.01
0.40	0.07	0.20	0.07	0.03
0.80	0.15	0.50	0.13	0.05
1.20	0.22	0.90	0.20	0.08
2.00	0.37	1.40	0.33	0.13
4.00	0.60	2.30	0.50	0.21
7.00	1.00	3.60	0.90	0.34
11.00	1.50	5.90	1.40	0.60
17.00	2.50	9.50	2.30	0.90
28.00	4.10	15.00	3.60	1.50
45.00	6.60	25.00	5.90	2.40
	11.00		9.50	3.80
	17.00		15.00	6.20
	28.00		25.00	10.00
	45.00			

Section1: Further in-depth analysis of the Slice & Dice python script

The input parameters in are the "AQ" time, a "Wait" time, the preferred ordering, and the 3 relaxation delay lists, or instructions on how to make the lists. The "AQ" time must be exactly or slightly longer than the time needed to collect one transient without a relaxation delay. The AQ is calculated by adding each part of the experiment, minus any relaxation period. For example, if we take the recovery delay as 1.5 s, each water suppression time is 80 ms and 40 ms (120 ms total), the direct acquisition time is 30 ms, and the transfers and indirect chemical shift evolution times are a maximum of 50 ms, we find a total of 1.8 s for each transient. The "Wait" time specifies the minimum amount of time between acquisitions for the purposes of limiting the probe duty factor. The program will stop after the first solution is found to (mostly) preserve the order of the delay lists. There are thus options to group the experiments together in different ways at the beginning of the calculation, *i.e.* grouping the smallest ^{13}C relaxation times together by alternating the $^{13}C^{ali}$ and $^{13}C'$ experiments, or grouping by the same type of experiment ($^{13}C^{ali}$ and $^{13}C'$ start separated). Finally, the delay schedules may be specified explicitly or automatically generated using a Fibonacci spacing with a minimum and maximum values and the number of points. Fibonacci spacing closely matches previously used delay schedules and is useful for when there is a large dynamic range in the relaxation rates in one measurement, such as in the $^{13}C^{ali}$ experiment. The ^{15}N list is initially arranged in reverse chronological order (largest time to smallest) and the ^{13}C experiments arranged chronologically. On the first pass, the program will fit as many ^{13}C experiments into the longest ^{15}N relaxation times as time allows. Once the ^{15}N delays can no longer accommodate the ^{13}C experiments, the inner and outer experiments are swapped, and the ^{15}N are fit inside the remaining ^{13}C delays. If a solution is not found on the first pass, the order of the relaxation times is varied until a solution is found. The order of the lists is varied using bubble sorts as follows: ^{15}N alone, then ^{15}N and $^{13}C'$ together, then ^{15}N and $^{13}C^{ali}$ together, and finally ^{15}N, $^{13}C'$and $^{13}C^{ali}$ altogether. If there are no solutions the lowest relaxation time of each list is removed and the search continues until a solution is found, or until there are only four items in the lists. If there are still no solutions, the best fit is reported. The output from the program prints the arrays and lists needed to modify the Bruker pulse program ("region", "Inner", "Outer", and the Actual Timings: "CA", "CO", "N"). An estimate is produced for the time required to collect 1 transient of the standard and embedded experiments to gauge the efficiency improvements. A "T_1" array is also produced that shows the estimated wait time between the final "Inner" loop acquisition and the "Outer" loop acquisition, where large values in the T_1 array may indicate inefficient packing. Changing either the "AQ" or "Wait" time slightly, or by adjusting the time scheduling slightly, may result in a more efficient use of time.

Table S2. Comparison of ^{15}N, $^{13}C'$ and $^{13}C^{\alpha}$ R_1 fits for individual residues of [U-^{1}H,^{13}C,^{15}N]GB1 crystal at 16.4 T and 100 kHz MAS with a nominal temperature of 282.1 K. The heavily overlapping peaks were eliminated from Fig. 2 but are retained in the table below for reference and highlighted in yellow. For each residue the white background denotes the rates acquired the traditional experiment, while the grey background indicates the Slice & Dice.

**Residue**	**^{15}N**		**$^{13}C'$**		**$^{13}C^{\alpha}$**	
	R_1 (10^{-2} s^{-1})	Error	R_1 (10^{-2} s^{-1})	Error	R_1 (10^{-1} s^{-1})	Error
**M01**	-	-	5.68	0.98	-	-
	-	-	7.06	0.96	-	-
**Q02**	7.97	0.87	-	-	3.57	0.29
	7.75	0.78	-	-	3.29	0.24
**Y03**	3.17	0.49	-	-	3.17	0.36
	3.20	0.36	-	-	3.22	0.32
**K04**	-	-	-	-	-	-
	-	-	-	-	-	-
**L05**	-	-	-	-	-	-
	-	-	-	-	-	-
**I06**	1.76	0.33	5.69	2.16	-	-
	1.30	0.34	5.03	2.07	-	-
**L07**	-	-	4.98	1.13	-	-
	-	-	6.11	1.20	-	-
**N08**	3.07	0.30	21.20	2.01	3.35	0.24
	3.43	0.34	20.85	2.14	3.40	0.20
**G09**	3.45	0.46	7.05	1.59	2.02	0.52
	3.34	0.45	10.48	1.69	2.41	0.48
**K10**	7.33	1.72	9.89	1.13	2.69	0.24
	6.81	1.73	10.28	1.20	2.64	0.17
**T11**	9.04	0.77	10.47	1.40	4.09	0.22
	7.93	0.69	12.29	1.33	3.91	0.20
**L12**	11.69	1.66	-	-	2.39	0.18
	10.44	1.71	-	-	2.28	0.21
**K13**	3.66	0.53	-	-	-	-
	3.34	0.44	-	-	-	-
**G14**	4.43	0.70	6.26	2.90	2.23	0.53
	4.74	0.74	4.69	1.73	2.30	0.50
**E15**	-	-	6.94	0.84	2.74	0.21
	-	-	6.49	0.68	2.90	0.18
**T16**	4.39	0.43	4.99	0.73	-	-
	3.26	0.35	5.11	0.66	-	-
**T17**	-	-	8.01	1.02	3.36	0.29
	-	-	6.89	0.72	3.21	0.24
**T18**	6.94	0.93	7.18	0.69	-	-
	5.97	0.77	7.62	0.67	-	-
**E19**	10.73	0.87	8.29	1.49	2.17	0.19
	9.55	0.75	8.15	1.16	2.12	0.17
**A20**	5.53	0.67	4.79	0.54	1.19	0.13
	4.53	0.54	5.31	0.62	1.30	0.12
**V21**	3.15	0.55	9.14	1.22	1.22	0.22
	3.85	0.46	10.21	1.49	1.25	0.19
**D22**	-	-	5.24	0.35	-	-
	-	-	5.69	0.79	-	-
**A23**	-	-	8.27	0.67	1.39	0.14
	-	-	7.85	0.60	1.49	0.13
**A24**	4.21	0.36	6.48	0.63	-	-
	3.87	0.36	6.60	0.67	-	-
**T25**	-	-	4.24	1.37	1.20	0.16
	-	-	3.77	1.02	1.30	0.16
**A26**	1.63	0.21	6.85	0.65	1.46	0.12
	1.53	0.23	7.43	0.59	1.28	0.12
**E27**	4.17	0.53	6.03	1.57	1.57	0.35
	5.01	0.62	9.19	1.83	2.04	0.35
**K28**	-	-	5.61	3.79	2.05	0.29
	-	-	4.91	1.38	2.40	0.30
**V29**	-	-	7.93	0.79	1.15	0.13
	-	-	9.22	0.83	1.25	0.12
**F30**	3.36	0.22	14.81	3.56	1.44	0.33
	3.20	0.29	9.50	2.56	1.99	0.33
**K31**	1.72	0.29	8.37	0.46	-	-
	2.11	0.37	8.61	0.44	-	-
**Q32**	2.43	0.47	10.20	1.11	2.02	0.22
	3.02	0.42	10.96	1.20	1.78	0.21
**Y33**	-	-	-	-	-	-
	-	-	-	-	-	-

**A34**	-	-	8.81	0.71	1.97	0.15
	-	-	8.18	0.58	1.96	0.14
**N35**	3.11	0.30	7.68	0.64	-	-
	2.94	0.27	8.39	0.60	-	-
**D36**	2.21	0.29	-	-	-	-
	2.16	0.33	-	-	-	-
**N37**	-	-	3.12	1.70	2.11	0.14
	-	-	4.09	1.76	1.92	0.13
**G38**	12.75	1.29	9.88	2.10	2.44	0.34
	10.79	1.15	9.92	1.74	2.67	0.36
**V39**	4.55	0.48	13.92	1.10	-	-
	3.85	0.39	12.79	1.18	-	-
**D40**	14.84	1.17	13.72	0.78	-	-
	14.39	1.16	11.71	0.71	-	-
**G41**	12.88	1.35	8.18	1.59	5.07	0.50
	15.34	1.73	6.89	1.09	5.71	0.47
**E42**	-	-	6.76	0.68	2.16	0.13
	-	-	6.73	0.67	2.40	0.16
**W43**	3.78	0.42	4.65	0.92	1.36	0.23
	3.52	0.38	5.94	0.92	1.29	0.21
**T44**	0.99	0.30	3.45	1.41	3.00	0.47
	1.33	0.30	5.21	1.09	2.43	0.43
**Y45**	1.32	0.29	3.46	1.21	1.61	0.32
	0.79	0.25	2.79	1.01	1.69	0.29
**D46**	3.12	0.45	-	-	3.57	0.39
	2.67	0.43	-	-	3.51	0.35
**D47**	3.89	0.55	9.63	1.76	-	-
	3.39	0.44	7.87	1.48	-	-
**A48**	-	-	10.75	1.64	2.24	0.12
	-	-	9.69	2.39	2.30	0.11
**T49**	5.26	0.53	5.80	1.16	-	-
	5.39	0.59	5.66	1.13	-	-
**K50**	3.21	0.76	5.45	1.19	-	-
	3.12	0.64	6.71	1.01	-	-
**T51**	2.06	0.30	4.96	0.81	2.23	0.21
	1.84	0.27	6.60	1.01	2.41	0.19
**F52**	1.25	0.25	3.27	1.61	2.64	0.52
	1.01	0.24	2.91	1.36	2.77	0.47
**T53**	1.06	0.28	7.81	1.60	3.91	0.40
	0.93	0.25	6.41	1.23	3.93	0.42
**V54**	1.03	0.19	7.65	1.27	1.33	0.24
	1.00	0.18	8.07	1.10	1.49	0.22
**T55**	1.61	0.25	7.09	0.50	-	-
	1.43	0.24	7.48	0.46	-	-
**E56**	4.18	0.49	-	-	-	-
	4.90	0.54	-	-	-	-

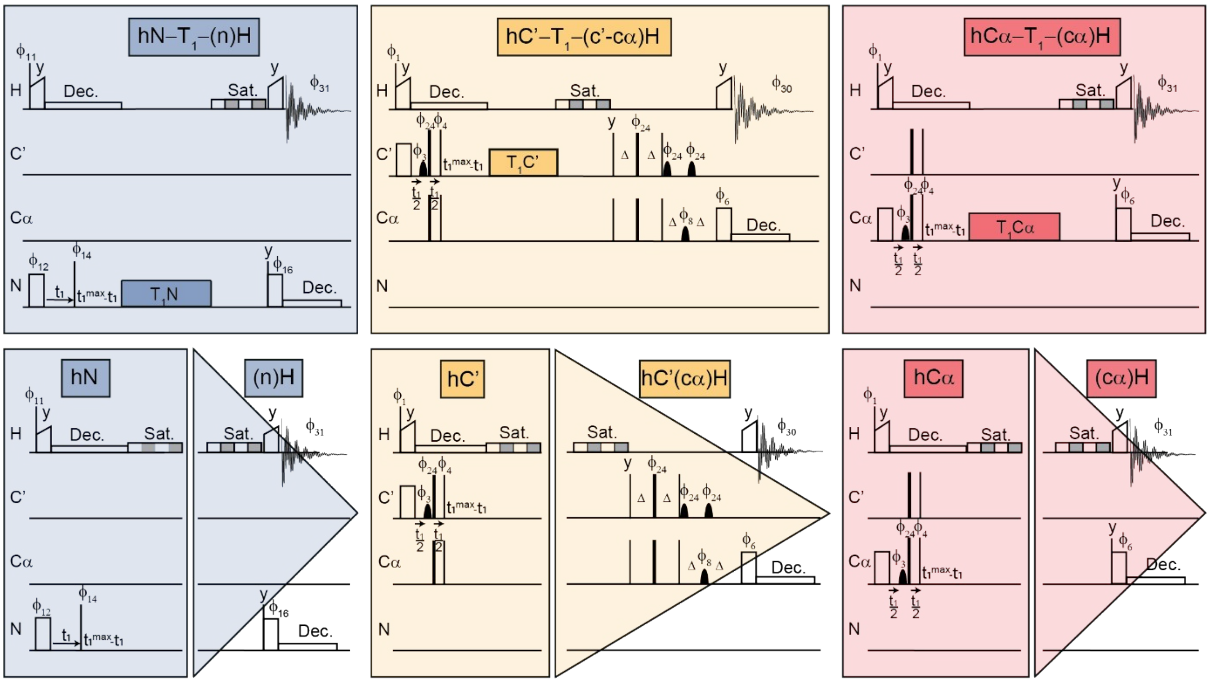

Figure S1. Pulse sequence of the individual ^{15}N (blue), ^{13}C' (yellow) and ^{13}C$^{\alpha}$ (red) R_1 measurements for the Slice & Dice experiment. Below each experiment is sliced into portions for excitation (square) and acquisition (triangle). Narrow and broad black lines represent 90˚ and 180˚ hard pulses, respectively. Rounded pulses represent 180° selective shaped pulses. When not shown, the phase of the pulses is x. The phase cycling for both the experiments is as follow.

hNH$_N$ experiment: φ_{11}= {x*2, -x*2}, φ_{12}= {x*4, -x*4}, φ_{14}= {y, -y}, φ_{16}= {x*8, -x*8} and acquisition φ_{31}= {y, -y, -y, y, -y, y, y, -y, -y, y, y, -y, y, y, -y, y, -y, -y, y}. States-TPPI is employed on φ_{14}.

hCαHα and hC'CαHα experiment: φ_1= {x*4, -x*4}, φ_3= {x*2, y*2}, φ_4= {y, -y}, φ_6= {x*8, -x*8}, φ_8= {x*16, y*16}, φ_{24}= {x, -x}. The acquisition is φ_{31}= {y, -y, -y, y, -y, y, y, -y, -y, y, y, -y, y, y, -y, y, -y, -y, y} for hCαHα, and φ_{30}= {y, -y, -y, y, -y, y, y, -y, -y, y, y, -y, y, y, -y, y, -y, -y, y, -y, y, y, -y, y, -y, -y, y, y, -y, -y, y, -y, y, y, -y} for hC'CαHα. States-TPPI is employed on φ_4. The MISSISSIPPI water suppression consists of four 10 ms periods with orthogonal phase with a saturation field of ~50 kHz (ν_1=ν_r/2).

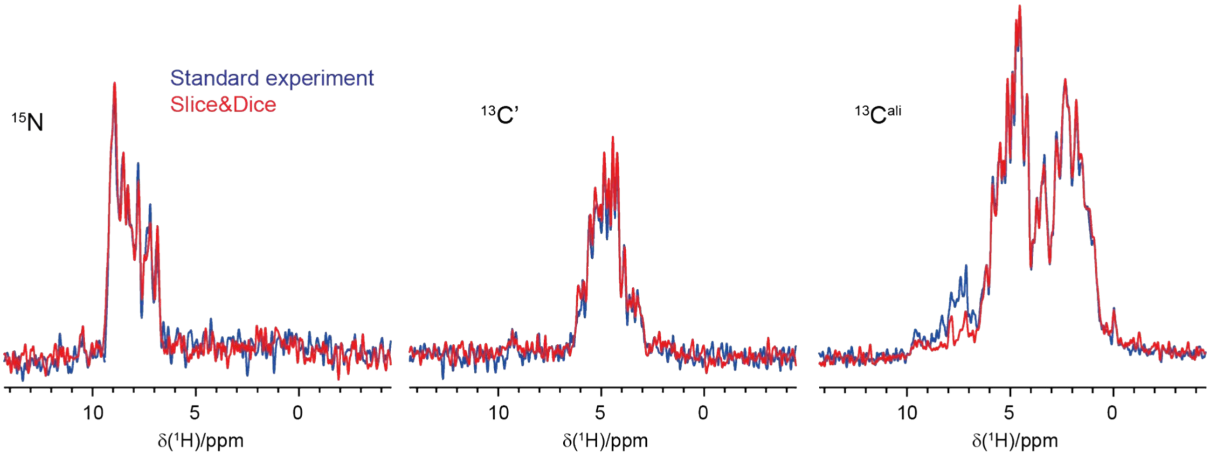

Figure S2. Sensitivity comparison of ^{1}H 1D integrated spectrum intensity on ^{15}N, ^{13}C' and ^{13}C^{ali} for the R_1 individual and the Slice & Dice experiment. Both implement a standard CP for all of the ^{1}H-X/Y transfer. The experiments were acquired with 32 coadded transients. The additional peaks left of the aliphatic region in the ^{13}C^{ali} standard experiment are due to aromatic residues that are excluded by a selective pulse in the Slice&Dice experiment.

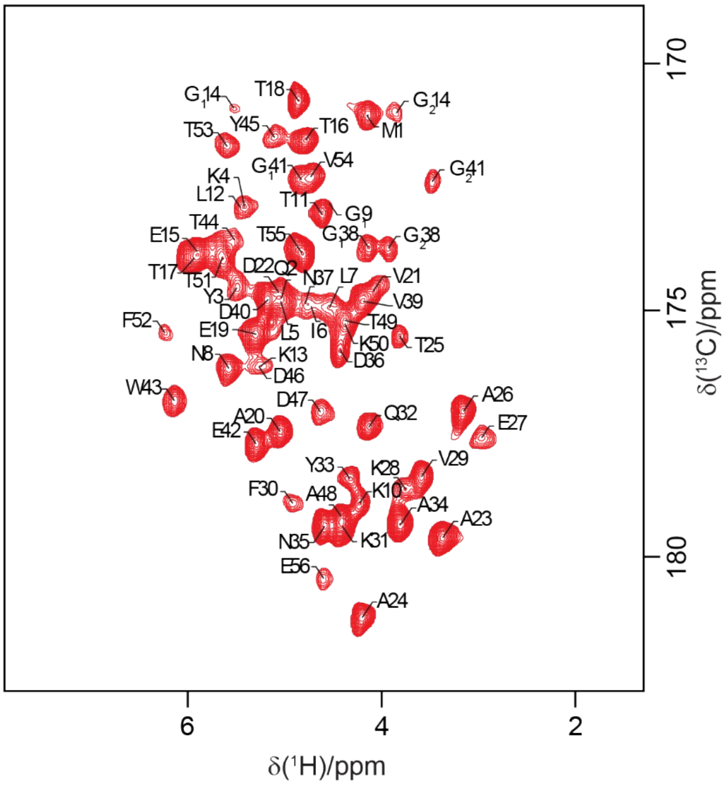

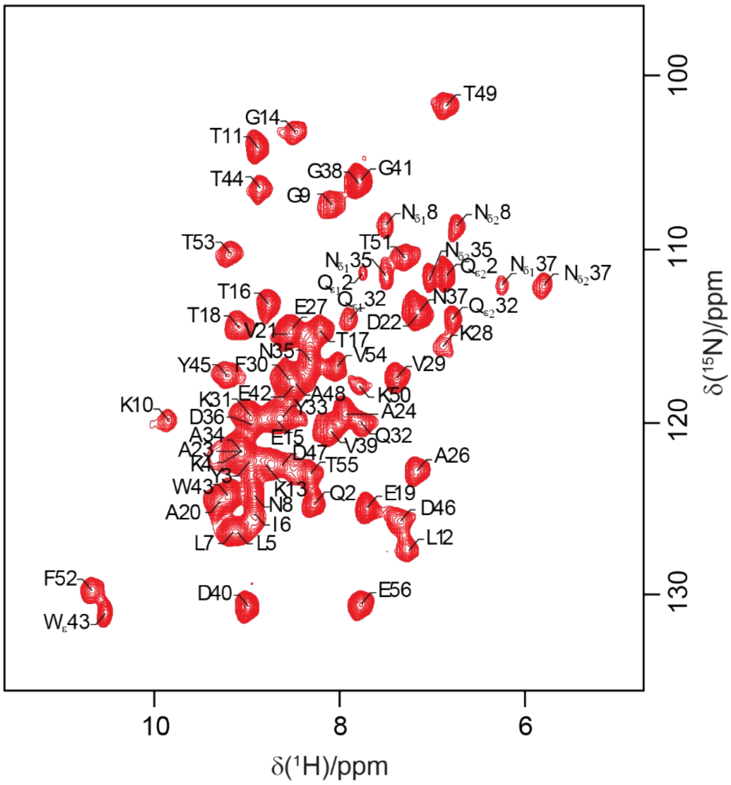

Figure S3. 2D spectra for crystalline [U-^{13}C,^{15}N]GB1 obtained at 100 kHz spinning using the Slice & Dice experiment with assignments of ^{13}C' (up) and ^{15}N (down) resonances.

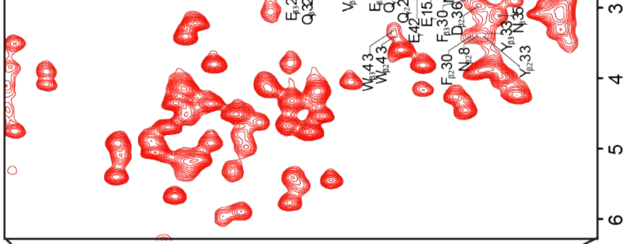

Figure S4a. 2D spectra for crystalline [U-^{13}C,^{15}N]GB1 obtained at 100 kHz spinning using the Slice & Dice experiment with assignments of the aliphatic ^{13}C. The spectrum is acquired folded and here is presented unfolded with a zoom on the α-region on the left, and of the rest of the ^{13}C^{ali} on the right, in which only assignments for non-overlapping peaks are shown for the spectrum. The contours for the aliphatic region are drawn lower than the ^{13}C^{α} region to highlight the number of resonances present.

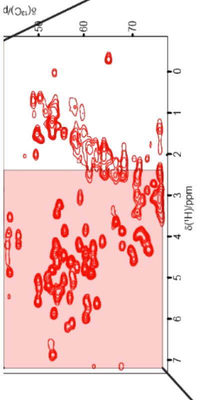

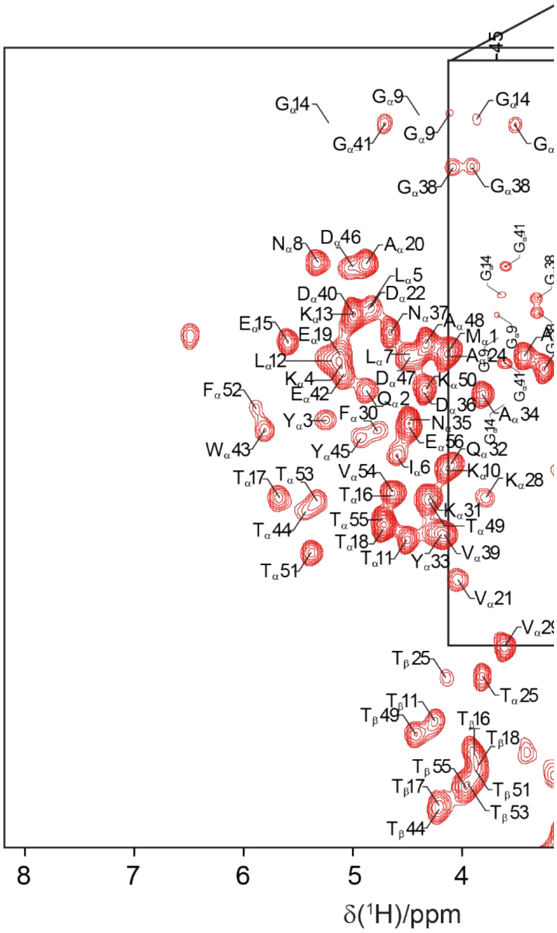

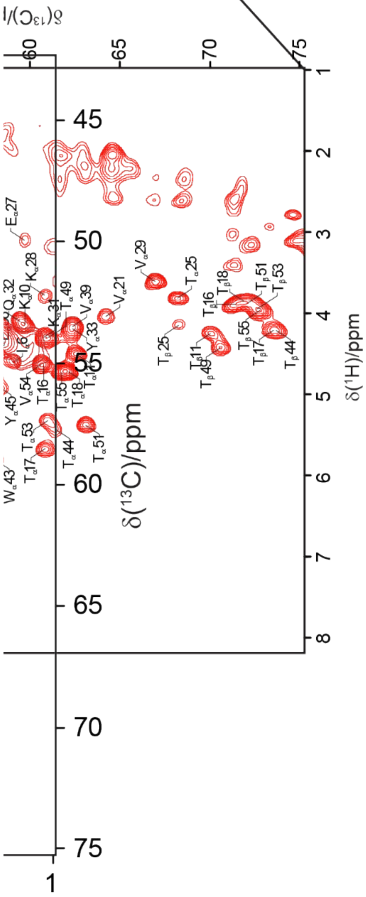

Figure S4b. Zoom of the 2D spectrum for the ^{13}C^{α}-region for crystalline [U-[illegible]ed at 100 kHz spinning using the Slice & Dice experiment (left spectrum in **Fig.S4a**).

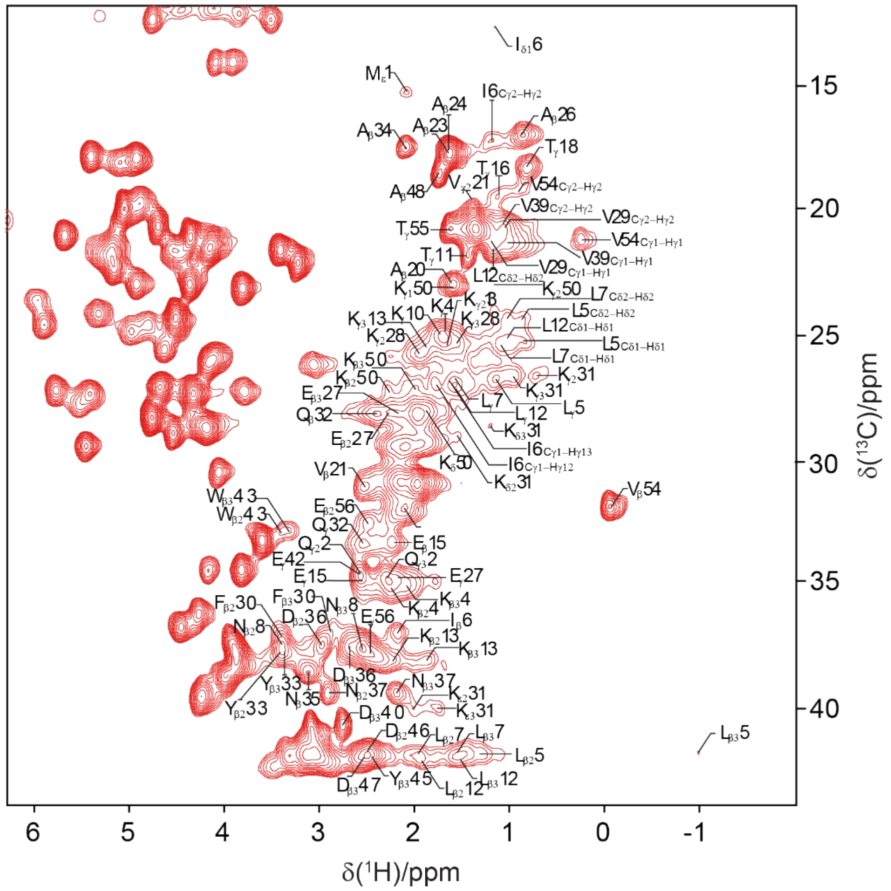

Figure S4c. Zoom of the 2D spectrum for the aliphatic carbon region for crystalline [U-^{13}C,^{15}N]GB1 obtained at 100 kHz spinning using the Slice & Dice experiment (right spectrum in **Fig.S4a).**

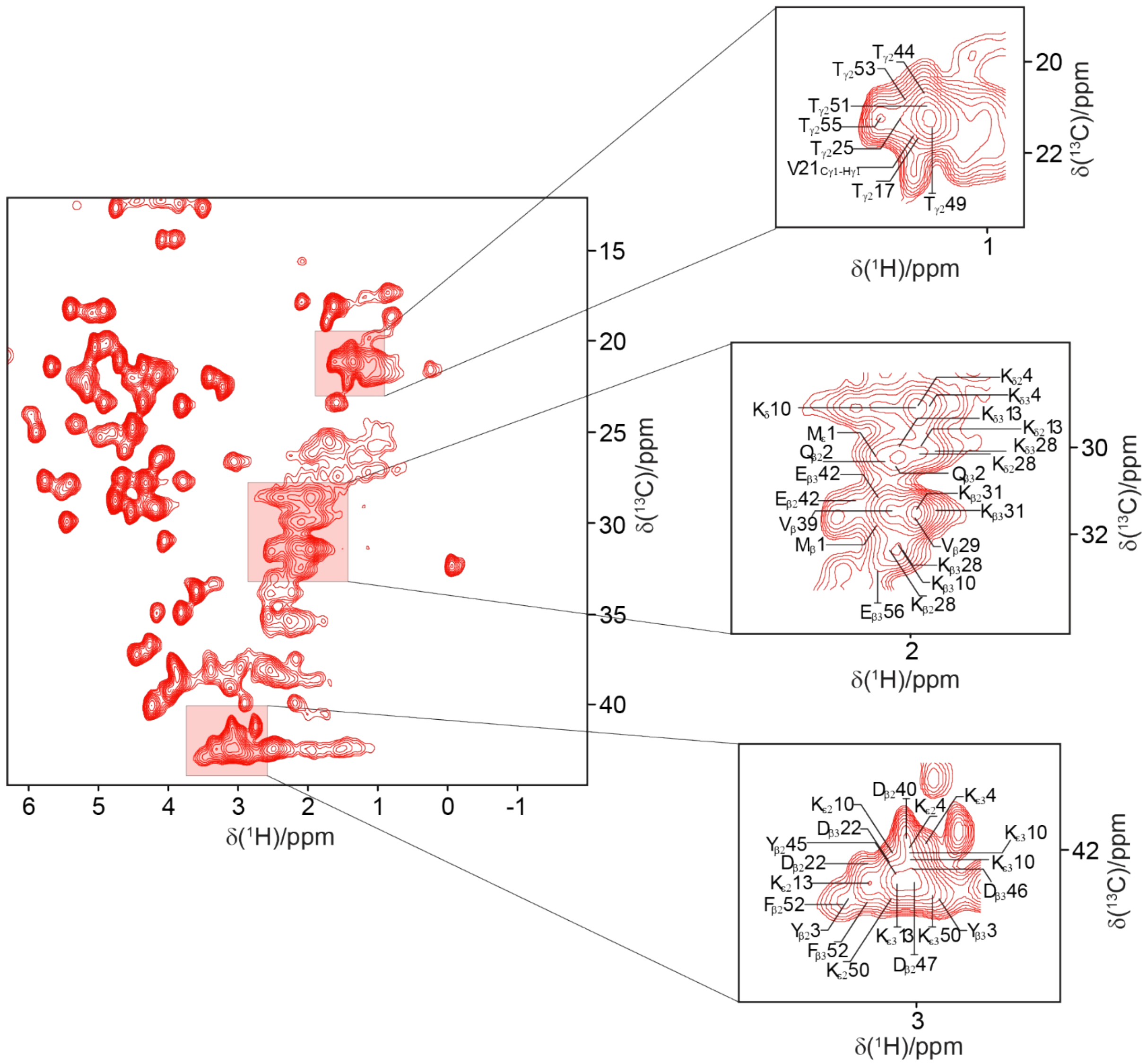

Figure S5. Expansion of the 2D spectrum for the aliphatic carbon region for crystalline [U-^{13}C,^{15}N]GB1 obtained at 100 kHz spinning using the Slice & Dice experiment with highlighted the overlapping resonances.

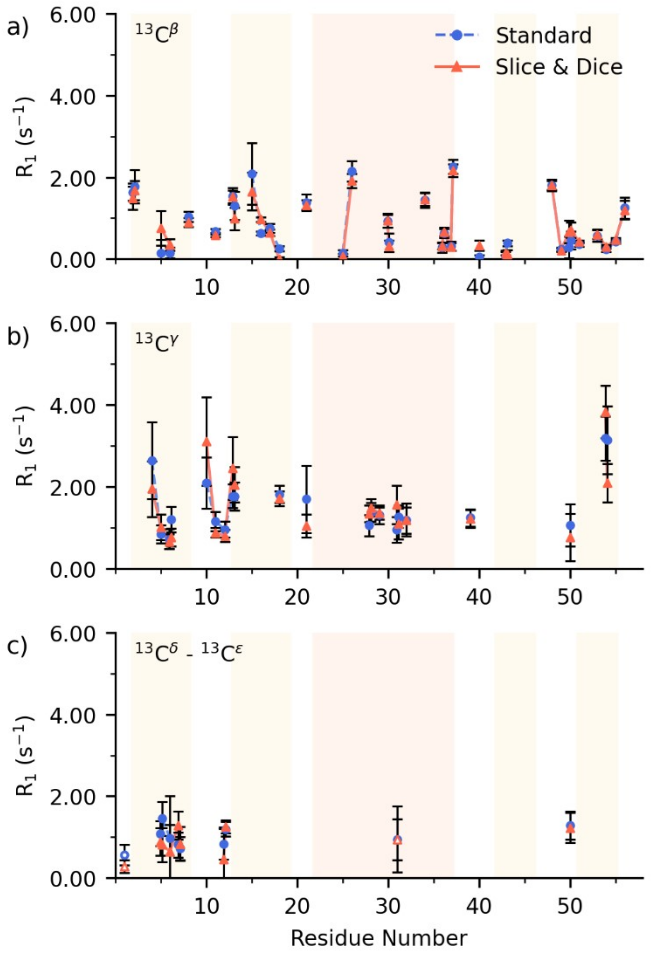

Figure S6. A comparison of the R_1 rates for a) ^{15}N, b) $^{13}C'$, c) $^{13}C^{\alpha}$ obtained from the separated single-acquisition experiment (filled-blue circle) and Slice & Dice (filled-red triangle) as a function of the residue number. In c) $^{13}C^{\delta}$ are indicated as described above and $^{13}C^{\varepsilon}$ are indicated with open-blue circle for standard acquisition and red-empty triangles for Slice & Dice. Errors bars represent two standard deviations within the correspondent rate. For the severely overlapping peaks, values are not included.

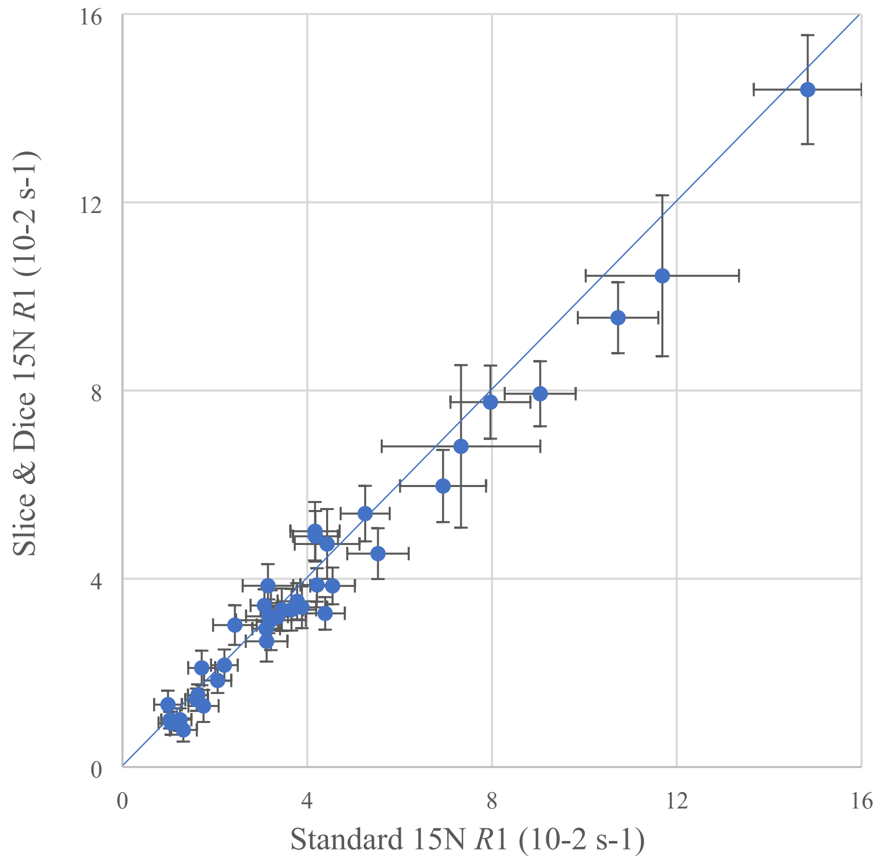

Figure S7. The ^{15}N R_1 rates measured by the standard experiment to the Slice & Dice approach in uniformly [^{1}H, ^{13}C, ^{15}N] labelled GB1. The error bars indicate two standard deviations.

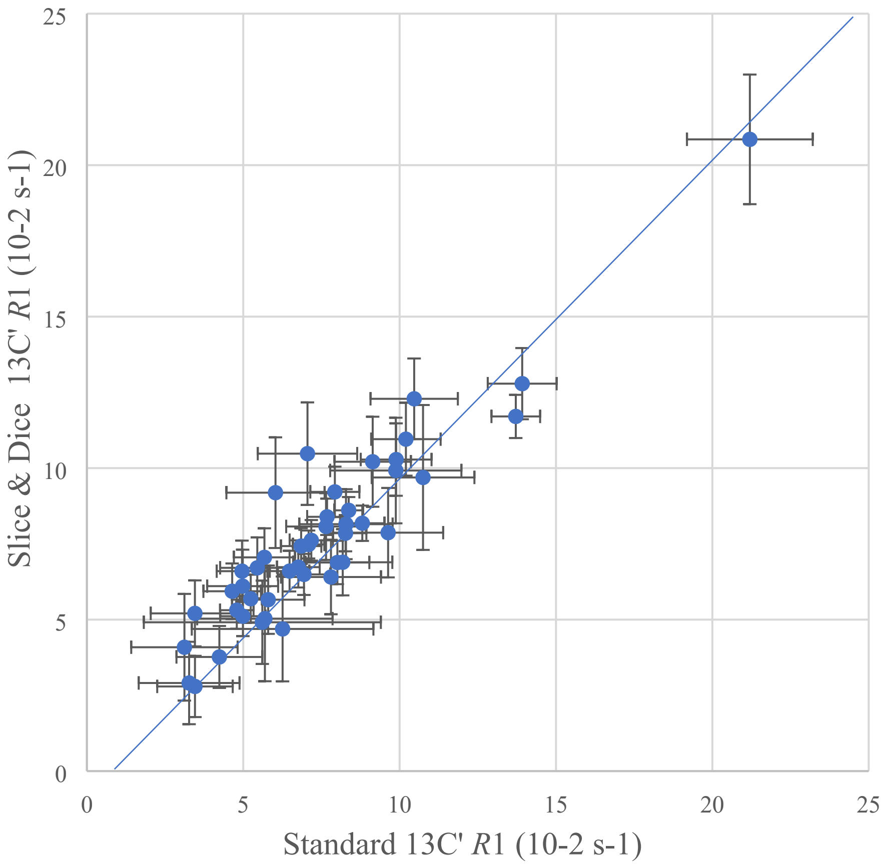

Figure S8. The $^{13}C'$ R_1 rates measured by the standard experiment to the Slice & Dice approach in uniformly [^{1}H, ^{13}C, ^{15}N] labelled GB1. The error bars indicate two standard deviations.

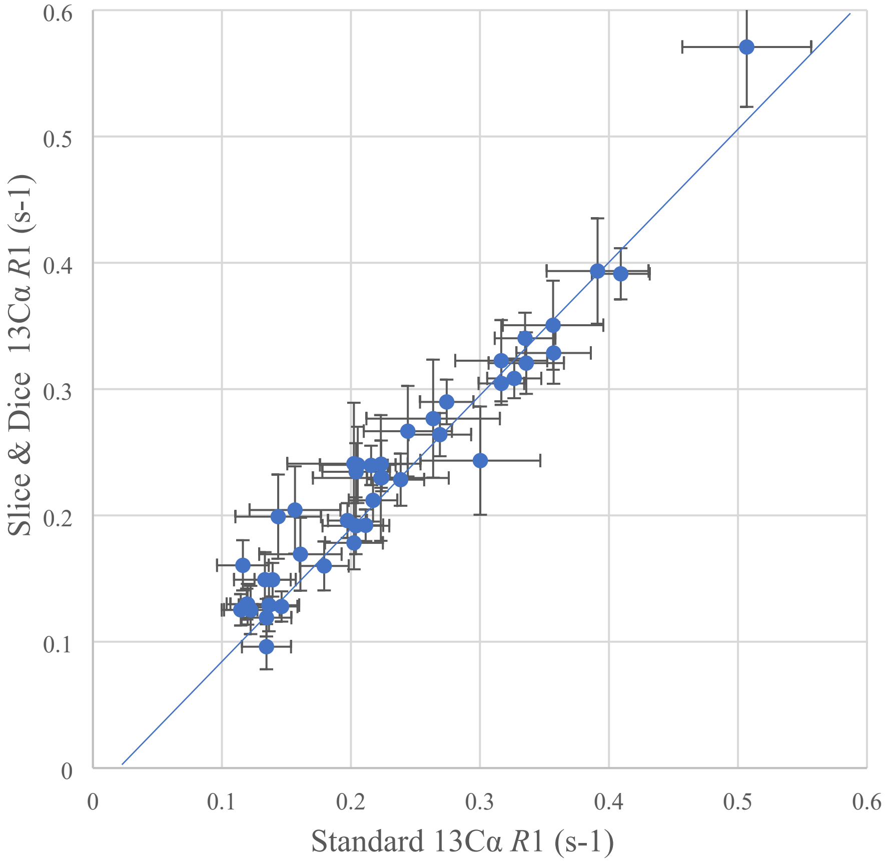

Figure S9. The $^{13}C^{\alpha}$ R_1 rates measured by the standard experiment to the Slice & Dice approach in uniformly [^{1}H, ^{13}C, ^{15}N] labelled GB1. The error bars indicate two standard deviations.

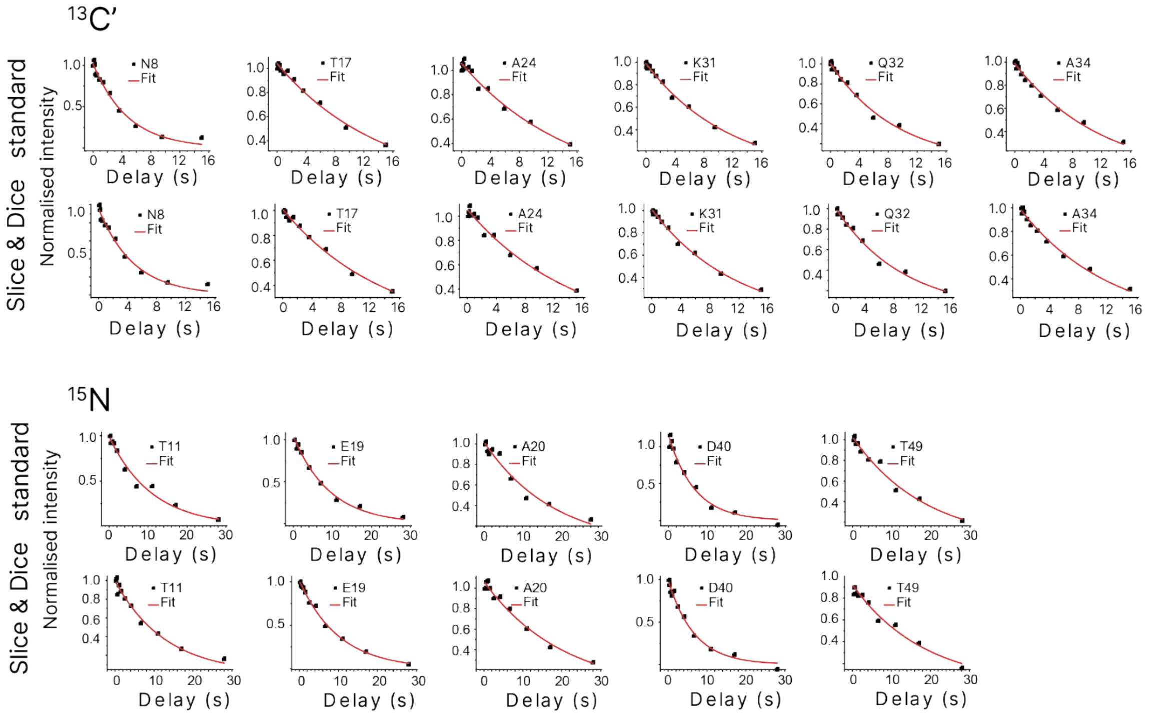

Figure S10. Comparison of selected representative relaxation curves and corresponding fits for $^{13}C'$ and ^{15}N measurements using standard and Slice & Dice approaches.
